# *Glycaspis brimblecombei* (Hemiptera: Psyllidae) attack patterns on different *Eucalyptus* genotypes

**DOI:** 10.7717/peerj.3864

**Published:** 2017-10-24

**Authors:** Juliana Tuller, Karla Nunes Oliveira, Jhonathan Oliveira Silva, Maurício Lopes de Faria, Mario Marcos do Espírito-Santo, José Eduardo Serrão, José Cola Zanuncio

**Affiliations:** 1 Departmento de Biologia, Setor de Ecologia e Conservação, Universidade Federal de Lavras (UFLA), Lavras, Minas Gerais, Brazil; 2 Pós-Graduação em Ecologia, Departamento de Biologia Geral, Universidade Federal de Viçosa, Viçosa, Minas Gerais, Brazil; 3 Colegiado de Ecologia, Universidade Federal do Vale do São Francisco (UNIVASF), Senhor do Bonfim, Bahia, Brazil; 4 Departamento de Biologia Geral, Universidade Federal de Viçosa (UFV), Viçosa, Minas Gerais, Brazil; 5 Departamento de Entomologia/BIOAGRO, Universidade Federal de Viçosa (UFV), Viçosa, Minas Gerais, Brazil

**Keywords:** Hybrid eucalypt, Biological control, *Psyllaephagus bliteus*, Red gum lerp psyllid, Water stress

## Abstract

**Background:**

The red gum lerp psyllid,* Glycaspis brimblecombei* Moore (Hemiptera: Psyllidae), an eucalypt insect pest from Australia, was reported in Brazil in 2003. This study evaluated damage patterns of this pest on *Eucalyptus camaldulensis* Dehn (Myrtaceae) and its hybrids *E. urophylla* X *E. camaldulensis* (urocam) and *E. urophylla* X *E. grandis* (urograndis). In addition, parasitism rates of *Psyllaephagus bliteus* Riek (Hymenoptera: Encyrtidae) on *G. brimblecombei* collected on different eucalypt genotypes are reported.

**Methods:**

Plantation plots of three eucalypt genotypes were evaluated over one year. The eucalypt leaves were collected and examined for attack by *G. brimblecombei*. Nymph parasitism of *G. brimblecombei* by *P. bliteus* was recorded.

**Results:**

Damage by *G. brimblecombei* was lower on the hybrid genotypes and on the adaxial surface of the eucalypt leaves. *G. brimblecombei* egg and nymph density were negatively correlated with monthly rainfall. Nymph parasitism of *G. brimblecombei* by *P. bliteus* was low (2.9%) independent of genotype and did not vary throughout the year.

**Discussion:**

Our data indicate the use of less susceptible eucalypt genotypes (e.g., hybrids) as an alternative to *G. brimblecombei* management. Because of the current low mortality rates for *G. brimblecombei* resulting from *P. bliteus* parasitism, biological control with this natural enemy is not recommended as a management strategy for *G. brimblecombei*.

## Introduction

The Australian genus *Eucalyptus* includes some of the most cultivated plants around the world. In Brazil, this genus was introduced in the second half of the 19th century (Couto & Betters, 1995), and its plantations cover more than 4.8 million ha (ABRAF, 2013). The *Eucalyptus* genus comprises more than 600 species (not including hybrids) with more than 30 species cultivated in Brazil (Queiroz, Burckhardt & Majer, 2012) for cellulose, charcoal, essential oils, furniture, planks, and paper production. *Eucalyptus* has a remarkable native and exotic phytophagous fauna in Brazil (Guedes et al., 2000), which can be explained by the suitable climatic conditions and its phylogenetic proximity to the Brazilian flora, with high Myrtaceae diversity (Zanuncio et al., 2001).

Exotic phytophagous insects causing economic losses in Brazilian eucalypt plantations (Pereira et al., 2001) include the blue gum lerp psyllid *Ctenarytaina eucalypti* (Hemiptera: Psyllidae), the eucalyptus snout beetles *Gonipterus gibberus* and *G. scutellatus*, the Australian eucalyptus longhorn *Phoracantha semipunctata* (Coleoptera: Cerambycidae) (Ribeiro et al., 2001; Queiroz-Santana & Burckhardt, 2007), the eucalyptus gall wasp *Leptocybe invasa* (Hymenoptera: Eulophidae) (Fernandes et al., 2014), and the Australian red gum lerp psyllid, *Glycaspis brimblecombei* Moore (Hemiptera: Psyllidae) (Ferreira-Filho et al., 2015). *G. brimblecombei* is the major problem in eucalypt plantations in Brazil and was first recorded in São Paulo State in 2003 (Wilcken et al., 2003), spreading quickly throughout the country (Pereira et al., 2013; Queiroz et al., 2013). Because *G. brimblecombei* can cause 20% to 30% of defoliation, crown thinning, and eucalypt mortality (Wilcken et al., 2003; Queiroz et al., 2013), there is an urgent need to develop strategies aiming to manage this pest in Brazil.

*G. brimblecombei* reproduces sexually, depositing 6–45 eggs per eucalypt leaf, preferentially on young leaves (Firmino-Winckler et al., 2009). Psyllid nymphs produce honeydew after initial feeding and use it with a wax secretion to build tapered protective white shelters (lerp) on the leaf surface (Sharma et al., 2013). *G. brimblecombei* has five nymph instars, with a complete lifecycle from 15 to 34 days, and several generations per year (Firmino-Winckler et al., 2009; Laudonia, Magiotta & Sasso, 2014). There are no records of diapause for this psyllid species, even during the winter in temperate countries (FAO, 2012; Laudonia, Magiotta & Sasso, 2014). In its original habitat in Australia, *G. brimblecombei* prefers to feed on *E. camaldulensis* Dehn (Myrtaceae) and natural enemies, such as parasitoids and predators, exert strong control of psyllid populations (Collett, 2001).

Integrated pest management (IPM) combines different control methods, such as physical, chemical (Zanetti et al., 2003), cultural, and biological methods (Grosman et al., 2005) to reduce pest damage (Kogan, 1998). However, IPM efficiency depends on understanding the pest lifecycle, susceptibility to environmental conditions, female preference and offspring performance on host plants and habitats, population dynamics, and mortality from natural enemies (VanLenteren et al., 2003; Lockwood & Gilroy, 2004; Pereira et al., 2013). Chemical control has low efficacy against *G. brimblecombei* (Queiroz-Santana & Burckhardt, 2007), but entomopathogenic fungi (DalPogetto et al., 2011), predators (Dias et al., 2012; Dias et al., 2014), and its main natural enemy from Australia, the parasitoid *Psyllaephagus bliteus* Riek (Hymenoptera: Encyrtidae), have been studied to manage this insect (Daane et al., 2005; Daane, Sime & Paine, 2012).

*P. bliteus* is a koinobiont parasitoid that prefers to oviposit on third-instar *G. brimblecombei* nymphs, but its offspring development is delayed until the psyllid reaches the fifth instar (Daane et al., 2005). The exoskeleton of the dead parasitized hosts forms an easily recognized mummy, and the psyllid nymph is completely consumed as the parasitoid nearly finishes development. The white *P. bliteus* larva can be seen throughout the mummified exoskeleton of *G. brimblecombei,* which becomes transparent (Sullivan et al., 2006). *P. bliteus* was accidentally introduced into Brazil and was first reported soon after its host in 2003 (Berti-Filho et al., 2003). Due to the low level of natural parasitism (0.2–11%) and to the reported success of this parasitod in controlling *G. brimblecombei* in the United States and Mexico, a program aimed at rearing and mass release of *P. bliteus* was started in Brazil (Ferreira-Filho et al., 2015). In Brazil, areas under this augmentative biological control showed a temporary increase in the parasitism rate of *G. brimblecombei* by *P. bliteus* up to 80%, although viable parasitoid populations were not found, requiring frequent releases (Ferreira-Filho et al., 2015).

The low long-term efficiency of *P. bliteus* to control *G. brimblecombei* through mass release programs indicate that integrative approaches are necessary, and the use of resistant eucalypt genotypes can be an alternative to pesticides for insect management. Hybrid eucalypt plants were developed for greater commercial value (such as wood density for furniture and construction and lignin content for charcoal) or increasing resistance to water stress and pests (Gonçalves et al., 2013). The susceptibility of *Eucalyptus* genotypes to insect species varies (Firmino-Winckler et al., 2009; Queiroz, Burckhardt & Majer, 2012) and plant mechanisms responsible for reducing damages by these organisms must be further investigated. Given that both temperature (Ferreira-Filho et al., 2015) and rainfall (Oliveira et al., 2012) affect *G. brimblecombei* abundance, further studies are necessary to determine of correct time of management interventions.

The present study aimed to evaluate the temporal and spatial patterns of abundance of *G. brimblecombei* on *Eucalyptus camaldulensis* and the hybrids *E. urophylla* X *E. camaldulensis* (“Urocam”) and *E. urophylla* X *E. grandis* (“Urograndis”), as well as the parasitism of this psyllid by the wasp *P. bliteus*, the following questions were addressed: (i) Do the densities of *G. brimblecombei* eggs and nymphs vary according to the host plant type, leaf surface (abaxial versus adaxial) and period of the year? (ii) Does the parasitism rate of *G. brimblecombei* nymphs by *P. bliteus* vary according to the aforementioned factors? (iii) Is the temporal variation of *G. brimblecombei* related to the amount of rainfall?

## Materials and Methods

### Study area

The study was conducted at Extrema farm (17°15′S 43°39′W), which is owned and managed by Vallourec & Mannesman Florestal S.A. in Olhos D’Água, Minas Gerais State, Brazil. This farm is located at an altitude of 800 m above sea level and has an area of 9,655.61 ha, of which 6,597.72 ha (68%) are planted with several different species and hybrids of *Eucalyptus*, and 1,884.22 ha (32%) are native vegetation remnants (mostly Cerrado fragments). This region has a tropical climate, with a dry season from June to September (Aw in Köppen’s classification), and a rainy season from November to March. The mean annual temperature is 24 °C and the total annual rainfall is 1,246 mm, according to data collected at a weather station at the Extrema farm. The selected plots had six year-old individuals of the river red gum *E. camaldulensis* Dehn, and two hybrids: *E. urophyla* × *E. camaldulensis* (urocam) and *E. urophyla* × *E. grandis* (urograndis).

### Sampling

Sampling was conducted in two plots per eucalypt plant genotype (*E. camaldulensis*, urocam and urograndis). Twenty eucalypt trees were randomly selected each month, from December 2006 to November 2007, and 10 leaves were collected per tree were individually bagged and taken to the laboratory. The number of *G. brimblecombei* eggs and nymphs at each instar were recorded for both the abaxial and adaxial leaf surfaces. The percentage of mortality inflicted by *P. bliteus* parasitism was calculated for nymphs between the third and the fifth instars by the following formula: (number of parasitized nymphs / total number of nymphs)*100. The viability of *G. brimblecombei* eggs was determined using the percentage of hatched eggs. After the insects were counted and removed, each leaf was scanned and its area was determined using the Image J software (Rasband, 2006). The density of *G. brimblecombei* eggs and nymphs per cm^2^ was determined for each leaf, and their mean densities were calculated per tree.

### Statistical analyses

Linear mixed effect models (LME) were constructed to determine the influence of *Eucalyptus* genotypes, leaf surface and time (explanatory variables) on the densities of *G. brimblecombei* eggs and nymphs and on the percentage of nymphs parasitized by *P. bliteus* (response variables). The effects of leaf surface on nymph density were only analyzed for *E. camaldulensis* because the number of nymphs was very low on urocam (45) and urograndis (48) genotypes. These models (one for each response variable) were tested against null models and followed by residual inspection of the error distribution. The LME models were used owing to random effects (Crawley, 2007), such as the nested structure of the data into different *Eucalyptus* genotypes/leaf surface. Time was only included as an explanatory variable in the models for *E. camaldulensis* because of the low density of *G. brimblecombei* on the two hybrid genotypes*.* Generalized linear models (GLM; one per response variable) were employed to test the influence of average monthly rainfall on the density of *G. brimblecombei* eggs and nymphs and on the percentage of nymphs parasitized by *P. bliteus*.

The non-significant variables were progressively (one-by-one) removed with the backward method, starting from the complete models containing all explanatory variables and their interactions until the minimal adequate models were obtained (Crawley, 2007). After this procedure, the differences between the levels of all categorical variables were tested with contrast analyses. All the models were also tested for the adequacy of the error distribution through residual analysis. The analyses were conducted with the software R version 2.14 (R Development Core Team, 2011).

## Results

**Figure 1:**
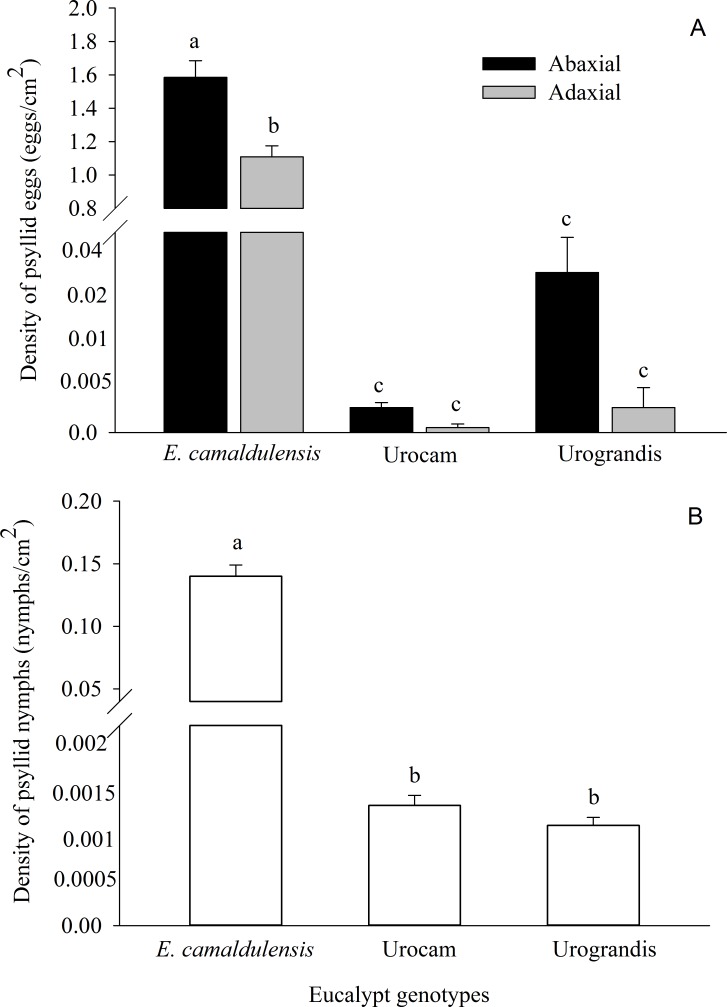
Mean density of (A) *Glycaspis brimblecombei* eggs on leaf surfaces of different eucalypt genotypes. (B) Mean density of *G. brimblecombei* nymphs (white columns) on leaves of different eucalypt genotypes. Error bars indicate standard errors. Different letters above the bars indicate statistically significant differences (*α* = 0.05).

A total of 481,212 eggs and 42,785 nymphs of *G. brimblecombei* were found on 14,388 eucalypt leaves. Egg and nymph densities were significantly higher on *E. camaldulensis* compared to the hybrids urograndis and urocam (Table 1; Fig. 1). Egg density also was higher on the abaxial surface of *E. camaldulensis* and urograndis, although this difference was only significant for *E. camaldulensis* (*p* < 0.001) (Fig. 1; Table 1). However, nymph density did not differ between surfaces for *E. camaldulensis* (*p* > 0.05). *G. brimblecombei* egg viability was 4%, 2% and 1% on *E. camaldulensis*, urocam and urograndis, respectively.

**Table 1:** Statistical parameters for the analyses evaluating *G. brimblecombei* attack patterns and its parasitism by *Psyllaephagus bliteus* on different *Eucalyptus* genotypes and leaf surfaces along twelve months. The effects of rainfall on monthly variations on psyllid egg and nymph density were also tested.

Response variable	Explanatory variable	*n*	*F*	*P*
Egg density	*Eucalyptus* genotype	1,140	537.90	<0.001
Nymph density	*Eucalyptus* genotype	1,140	250.00	<0.001
Percentage of parasitized nymphs	*Eucalyptus* genotype	1,140	0.24	0.665
Egg density on *E. camaldulensis*	Leaf surface	480	21.77	<0.001
Egg density on urograndis	Leaf surface	480	0.83	0.363
Egg density on urocam	Leaf surface	480	0.02	0.878
Nymph density *E. camaldulensis*	Leaf surface	480	0.72	0.464
Egg density on *E. camaldulensis*	Time (month)	1,440	51.49	<0.001
Nymph density on *E. camaldulensis*	Time (month)	1,440	85.57	<0.001
Percentage of parasitized nymphs	Time (month)	1,440	1.47	0.128
Egg density on *E. camaldulensis*	Monthly rainfall	12	67.43	<0.001
Nymph density on *E. camaldulensis*	Monthly rainfall	12	83.48	<0.001

We found 976 nymphs of *G. brimblecombei* parasitized by *P. bliteus* on *E. camadulensis*, but the mortality caused by this parasitoid was low (2.28%). Only one and two nymphs were parasitized on urograndis (2.08%) and urocam (4.44%), respectively, because the availability of 3–5th instar *P. bliteus* nymphs on these genotypes was very low (Fig. 1B).

**Figure 2:**
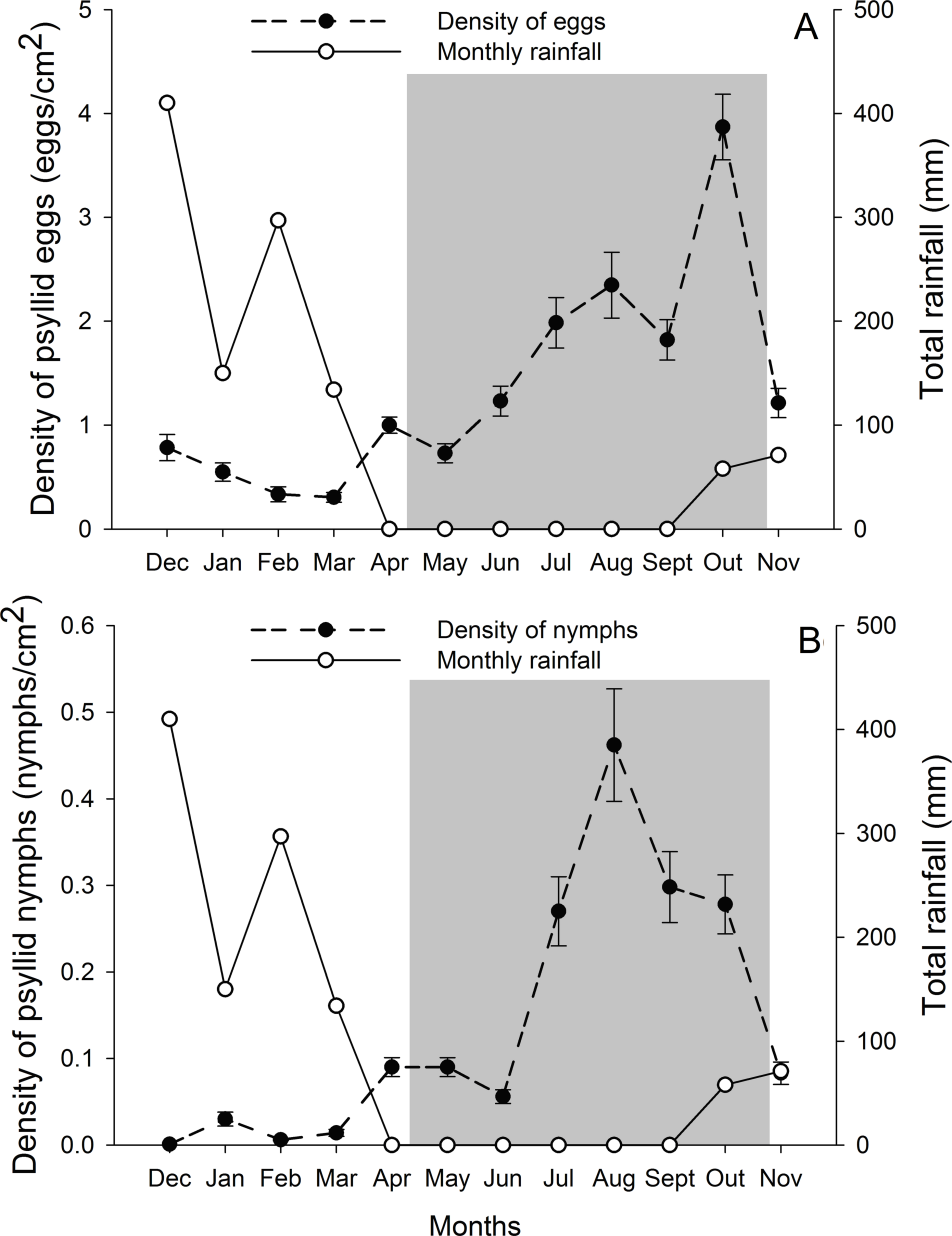
Monthly total rainfall and temporal variation on the mean number of *G. brimblecombei* eggs (A) and nymphs (B) on *E. camaldulensis* from December 2006 to November 2007. Error bars indicate standard errors. The shaded area indicates the dry season.

**Figure 3:**
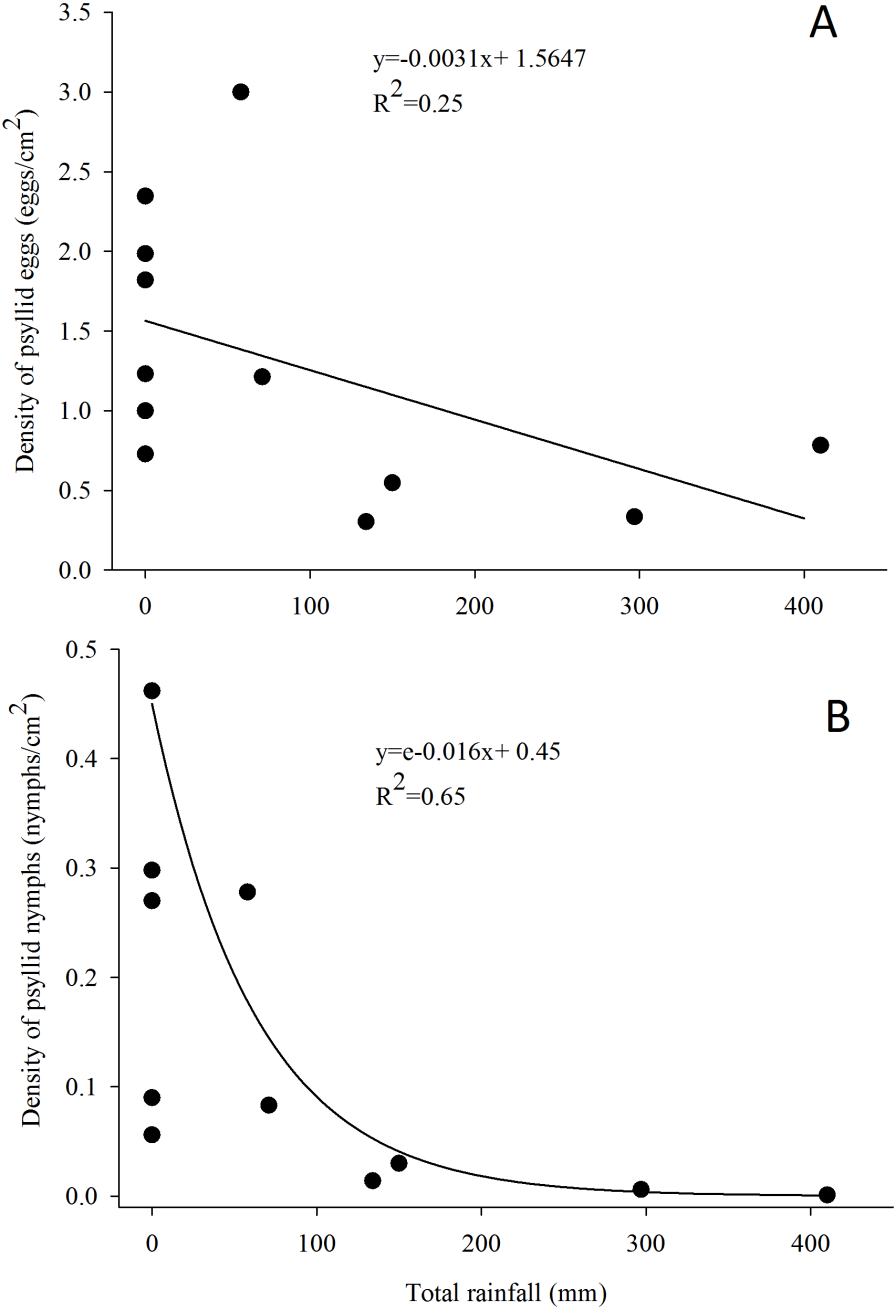
Relationship between the density of *G. brimblecombei* eggs (A) and nymphs (B) on *E. camaldulensis* and monthly total rainfall (*n* = 12). The estimated parameters were used to fit the curve obtained with the minimal adequate GLM model, in order to adjust linear and exponential functions in figures (A) and (B), respectively.

*G. brimblecombei* attacks on *E. camaldulensis* varied throughout the year, with egg and nymph densities of this insect peaking during the dry season (May to October) and decreasing in the rainy season (December to March) (Table 1; Fig. 2). Egg and nymph density were negatively correlated with monthly rainfall (Table 1; Fig. 3).

## Discussion

We detected marked spatial and temporal patterns of attack in different *Eucalyptus* genotypes by *G. brimblecombei* during the study period. Both the preference (as indicated by egg density) and performance (percentage of eggs hatched) were higher on *E. camaldulensis* than hybrids. At a finer scale, psyllid females preferred to lay their eggs on the abaxial surface of eucalypt leaves. The strong temporal variation on the abundance of *G. brimblecombei* highlights the susceptibility of this insect to climatic conditions. These results, coupled with the with low parasitism rates by *P. bliteus* detected in this psyllid population, can improve IPM strategies for a more effective control of *G. brimblecombei* on eucalypt plantations.

The higher *E. camaldulensis* susceptibility to the red gum lerp psyllid corroborates the field and laboratory studies on this pest in Brazil (Wilcken et al., 2003; Pereira et al., 2013) and other parts of the world (Wilcken et al., 2003; Valente & Hodkinson, 2009). The oviposition preference of *G. brimblecombei* females for *E. camaldulensis* is linked to higher egg viability and nymph survival on this eucalypt species (Firmino-Winckler et al., 2009). Nevertheless, the mechanisms responsible for such differential preference and performance between *E. camaldulensis* and hybrid lineages are still poorly studied. One hypothesis is that the long coexistence of *E. camaldulensis* and *G. brimblecombei* in Australia (Phillips, 1992) favors the successful attack on the natural genotype, possibly because this insect evolved the capacity to deal with chemical and physical defenses of *E. camaldulensis* leaves. Thus, studies involving leaf morphology and physiology are needed to identify the resistance mechanism of hybrid *Eucalyptus* genotypes for *G. brimblecombei*. Interspecific and phenotypic differences on leaf traits (e.g., texture, roughness, and trichome density) occur between *Eucalyptus* genotypes (Reifenrath, Riederer & Müller, 2005) and may play an important role in psyllid preference and performance, especially affecting the adhesion of first-instar nymphs to the leaf surface. Higher lerp abundance and lower *G. brimblecombei* nymph and adult mortality on *E. globulus* leaves were observed when epicuticular wax was removed (Brennan & Weinbaum, 2001a). The amount of epicuticular wax varies among *Eucalyptus* genotypes and is important for reducing the adhesion of psyllid nymphs (Brennan & Weinbaum, 2001a) and stylet probing (Brennan & Weinbaum, 2001b) on waxy, resistant eucalypt leaves.

As a whole, females preferred to oviposit on the abaxial surface of the three eucalypt genotypes, although statistically significant differences were only detected for *E. camaldulensis*. This pattern was already observed in previous studies with *G. brimblecombei* (Firmino-Winckler et al., 2009; Oliveira et al., 2012) and was explained by the higher nutrient flow for nymph development and reduced desiccation on the abaxial surface, especially during insect molt (Phillips, 1992; Firmino-Winckler et al., 2009). Thus, a preference-performance link would be expected, with higher nymph density on the abaxial surface. This was not the case for *E. camaldulensis,* but the underlying mechanisms are yet to be determined. Since parasitism levels did not differ between leaf surfaces, it is possible that first-instar nymphs emerging from eggs on the abaxial surface migrate to the adaxial surface to reduce intraspecific competition. Observational studies on nymph behavior would help understand this small-scale distribution pattern of *G. brimblecombei* on *E. camaldulensis.*

The low parasitism levels of *G. brimblecombei* by *P. bliteus* observed in our site corroborate other studies under natural conditions conducted in Brazil (0.2–11%; Ferreira-Filho et al., 2015) and United States (1.67–33%; Daane, Sime & Paine, 2012). In several countries, rearing and mass release of *P. bliteus* showed promising results, increasing parasitism levels in the field up to 94% (Huerta, Faundez & Araya, 2010; Ferreira-Filho et al., 2015). However, this parasitoid failed to establish viable populations in regions of warm climate, such as some parts of California (Daane, Sime & Paine, 2012) and in Brazil (Ferreira-Filho et al., 2015). Thus, it is likely that an effective control of *G. brimblecombei* using *P. bliteus* would demand periodic mass releases, increasing the financial costs of this strategy.

The peak in the egg and nymph density of *G. brimblecombei* on *E. camaldulensis* during the dry season corroborates other studies conducted for this species in Brazil (Wilcken et al., 2003; Ferreira-Filho et al., 2015). Several studies conducted with *G. brimblecombei* in other countries indicate that temperature is the main factor involved in population dynamics of this psyllid (Paine, Millar & Hoddle, 2000; Ramirez, Mancera & Guerra-Santos, 2003; Laudonia, Magiotta & Sasso, 2014). Under laboratory conditions in Brazil, Firmino found that 26 °C is the optimal temperature for the development and reproduction of *G. brimblecombei*. Thus, in our study site, the temperature was adequate for this species during the entire year and was likely not driving temporal variations in the abundance of *G. brimblecombei*. Instead, our results clearly indicate a strong effect of rainfall on the egg and nymph density, a pattern already described for *G. brimblecombei* in Mexico (Ramirez, Mancera & Guerra-Santos, 2003) and Mauritius Islands (Sookar, Seewooruthun & Ramkhelawon, 2003). Furthermore, a controlled experiment of rainfall simulation showed that the mechanical removal of the psyllid lerps by water droplets and/or lerp solubilization by leaf moisture may decrease its population (Oliveira et al., 2012). In addition, the higher humidity during the rainy season increases entomopathogenic fungi occurrence, which may kill psyllid nymphs (Ramirez, Mancera & Guerra-Santos, 2003). Long-term studies are needed to confirm the temporal pattern described here and assess other potential mechanisms driving the abundance of *G. brimblecombei*, such as physiological changes induced by water stress during the dry season.

This is one of the few studies addressing spatial and temporal patterns of abundance of *G. brimblecombei* and its levels of mortality using multiple eucalypt genotypes under field conditions. Despite the temporal variations on psyllid density and need for confirmation through long-term sampling, our one-year study is the longest conducted until now with this species, and confirms other results that indicate the higher abundance of *G. brimblecombei* during the dry season in Brazil. Thus, management strategies should consider the synchronization of planting with the rainy season to avoid the exposition of susceptible saplings of *Eucalyptus* spp. to severe attack by *G. brimblecombei*. Our findings indicate that using resistant *Eucalyptus* hybrids such as urocam and urograndis is a better management option than relying on *P. bliteus* to control the damage caused by the psyllid, due to its low parasitism levels. Studies aimed at determining leaf traits correlated to hybrid resistance to *G. brimblecombei* are necessary to select other commercially viable eucalypt genotypes or to enhance these traits through genetic improvement of susceptible species.

##  Supplemental Information

10.7717/peerj.3864/supp-1 Data S1 Dataset

## Additional Information and Declarations

### Data Availability

The following information was supplied regarding data availability: The raw data has been supplied as Data S1.
